# Immune checkpoint blockade induced shifts in cytokine expression patterns in peripheral blood of head and neck cancer patients are linked to outcome

**DOI:** 10.3389/fimmu.2023.1237623

**Published:** 2023-10-02

**Authors:** Louisa Röhl, Jana Wellhausen, Michael Berszin, Irene Krücken, Veit Zebralla, Markus Pirlich, Susanne Wiegand, Andreas Dietz, Theresa Wald, Gunnar Wichmann

**Affiliations:** ^1^ Department of Otorhinolaryngology, Head and Neck surgery, University Hospital Leipzig, Leipzig, Germany; ^2^ Institute of Pathology, University Hospital Leipzig, Leipzig, Germany

**Keywords:** immune checkpoint inhibitor-blockade (ICB), programmed-death 1 (PD-1), head and neck squamous cell carcinoma (HNSCC), liquid biopsy, cytokine expression pattern (CEP), interferon gamma (IFN-γ), outcome research, overall survival

## Abstract

**Background:**

Immune-checkpoint blockade (ICB) of programmed-death-1 (PD-1) with pembrolizumab or nivolumab is approved for treating recurrent/metastatic (R/M) head and neck squamous cell carcinoma (HNSCC). NadiHN and ADRISK are phase IIB trials investigating in locally advanced (LA) HNSCC having low or high risk of recurrence the potential benefits from adding nivolumab to post-operative radiotherapy or pembrolizumab to cisplatin-based radio-chemotherapy.

**Methods:**

Along five randomized controlled ICB trials including NadiHN and ADRISK, blood samples were taken before and after starting ICB in *n*=25 patients. Concentrations of vascular endothelial growth factor A (VEGF), CCL2 (MCP-1), interleukin-6 (IL-6), IL-8, interferon-gamma (IFN-γ), and CXCL10 (IP-10) pre- and post-ICB in EDTA-anticoagulated plasma and serum were compared. We used receiver operating characteristic (ROC) curves to identify optimal cutoff for defining subgroups before analyzing overall survival (OS) applying Kaplan–Meier plots and multivariate Cox regression.

**Results:**

We detected huge heterogeneity between cytokine patterns in pre-and post-ICB plasma and serum. We observed high correlation between concentrations of some cytokines. Despite absent systematic OS differences after ICB with pembrolizumab or nivolumab or between LA-HNSCC versus R/M HNSCC patients, we noticed improved outcome of patients having lower IFN-γ concentrations pre- and post-ICB and following ICB reduced concentrations of VEGF, IL-6, and IL-8 but not MCP-1. Contrarily, increases in IL-6, IL-8, and VEGF levels correlated with impaired outcome. Multivariate Cox regression revealed five independent OS predictors among cytokines; using natural logarithms of their hazard ratios to estimate an individual’s risk of dying, three cytokine-expression pattern (CEP)-risk groups with no death within mean (95% confidence interval) follow-up of 29.2 (22.1–36.2) months and median OS of 11.3 (8.8–13.8) and 2.9 (0.4-5.4) months were found.

**Conclusion:**

Whereas individual pre- or post-ICB cytokine concentrations in serum or plasma alone failed to predict the survivor group, CEP-risk groups may support the identification of individual patients with long-lasting benefit from ICB.

## Introduction

The tumor immune microenvironment (TIME) reflects ongoing immunological processes and is of central importance for either immune tolerance and proangiogenic growth support or suppression and elimination of neoplastic transformed cells. Tumors employ various mechanisms to escape immune surveillance and antigen-specific (adaptive) attack by tumor-infiltrating cytotoxic T cells (Tc) or natural killer (NK) cells belonging to the innate immune system. While NK cells respond to various NK cell receptors, Tc detect tumor-associated antigen (TAA)-derived peptides presented in proteins belonging to the major histocompatibility complex (MHC), the so-called human leukocyte antigen (HLA) proteins in man. Tc and NK responses, however, not only depend on ligand–receptor interaction of surface membrane receptors with surface–membrane receptors on tumor cells. In the TIME, interactions of Tc and antigen-presenting cells (APC) and Tc and NK cells with tumor cells are orchestrated by cytokines and other soluble factors. These soluble factors either stimulate or suppress particular signaling pathways. This contributes to either an effective anti-tumoral immune response eliminating the tumor or adaptive immune resistance (AIR), allowing for immune escape. One well-known AIR mechanism contributing to AIR is the response of tumors to an ongoing immune response through the induction of CD274 (PD-L1) expression through interferon-γ (IFN-γ) (1, 2). This is important, as increased PD-L1 expression allows PD-L1 binding to programmed-death-1 (PD-1) expressed on Tc and NK cells and consequently inhibits their anti-tumoral activity (3).

Pembrolizumab (Keytruda^®^, MK-3475, Merck Sharp & Dohme Corp, Whitehouse Station, USA) and nivolumab (Opdivo^®^, BMS-936558-01, Bristol-Myers Squibb Company, New York City, USA) are humanized monoclonal antibodies targeting the PD-1 protein and blocking the binding of PD-L1. They are currently under investigation in randomized controlled trials (RCTs) in head and neck squamous cell carcinoma (HNSCC) either alone or in combination with other chemotherapeutic drugs. Both immune checkpoint inhibitors have led to promising therapy regimens in the treatment of various malignancies including HNSCC (4). Following the results of the open-label, multicenter, phase IB trial KEYNOTE-012 and the randomized open-label, phase III study KEYNOTE-48, pembrolizumab received approval for the first-line treatment of recurrent and/or metastatic HNSCC (R/M HNSCC) (5–8). The ADRISK study, a multicenter randomized phase II study, is currently investigating if the addition of pembrolizumab to postoperative adjuvant radio-chemotherapy (aRCH) with cisplatin can improve the event-free survival (EFS) compared with aRCH alone in locally advanced intermediate and high-risk HNSCC (ClinicalTrials.gov NCT03480672). Nivolumab was approved for the treatment of R/M HNSCC after progress on standard-of-care platinum-based therapy following the multicenter randomized open-label phase III CheckMate-141 trial (9). The NadiHN trial, an open-label randomized phase II trial, investigating the response of intermediate-risk HNSCC patients after surgery to treatment with nivolumab plus radiotherapy versus radiotherapy alone stopped further accrual of patients (EudraCT No. 2016-004787-20).

Even though several studies confirm the efficacy of pembrolizumab and nivolumab in recurrent HNSCC, anti-PD-1 antibodies are not effective in every patient (10, 11). There is neither a definitive biomarker that allows sufficient patient selection nor one that allows distinction between a non-responder experiencing progress and a responder with prolonged overall survival (OS). Therefore, a definitive biomarker is needed to guide patient selection and to provide early on-treatment indicators of response, but none is available until now (12).

As stated above, PD-L1 overexpression can promote immune evasion and is found in over 55% of HNSCC (13, 14). This indicates that PD-L1 expression is a mechanism for cancer to escape immune-mediated destruction (15), and PD-L1 expression on tumor and/or immune cells is associated with a better response to anti-PD-1 therapy (10, 13, 16).

Taking the percentage of PD-L1 expression of tumor cells and immune cells into account, the tumor-positivity score (TPS) and immune score (IC) can be determined and a combined positive score (CPS) calculated to describe the PD-L1 status in HNSCC (14) that is linked to differences in response and survival of patients (8, 17, 18). This is reflected in the approval of pembrolizumab only for the treatment of R/M HNSCC with TPS ≥50% or CPS ≥1 (5–8). However, there are non-responders within CPS ≥ 1 and responders within CPS < 1 HNSCC patients (5–8, 17, 18). Therefore, other biomarkers that are not solely linked to IFN-γ signaling might be essential contributors to AIR.

Cytokines, chemokines, growth factors, and other soluble signaling proteins are produced not only by many immune cells but also endothelial and epithelial cells. They are pleiotropic stimulators or regulators of immune responses and have high biological activity (19, 20). Therefore, their concentration in body fluids, e.g., blood, in healthy individuals is mostly present at pg/ml concentrations (21). During the activation of a cytokine pathway associated with inflammation or disease progression, their concentration can increase up to 1,000-fold (22). The role of cytokines as potential biomarkers in cancer has been investigated by many studies (23–26) including our group (27, 28). Some cytokines are excessively produced by tumor cells (29), and consequently, varying cytokine concentrations seem to be valid predictors for disease progression and the effects of treatment (30, 31). Dysregulated expression of cytokines and chemokines and their receptors is a hallmark of many cancers, including HNSCC (32). However, the same is true for growth factors and their receptors, for instance the vascular endothelial growth factor A (VEGF) and others. We recently demonstrated and validated that pre-therapy VEGF plasma concentrations are an independent predictor of outcome in HNSCC (27). VEGF expression in HNSCC correlates with the expression of particular cytokines (28) *ex vivo* and in the TIME and contributes to malignant pathogenesis, and changes in pre- and post-treatment levels of these cytokines have been evaluated as markers for treatment outcome (33, 34). A decrease in plasma interleukin 6 (IL-6) levels correlated with improved PFS in NSCLC patients under anti-PD-1 therapy (35). Low serum levels of IL-6 and VEGF were associated with better clinical outcomes in HNSCC patients treated with cetuximab (26). Lower VEGF levels were also identified to be prognostic biomarkers in HNSCC patients among multiple cohorts (27, 36). Interleukin-8 (CXCL8, IL-8) is mainly produced by tumor cells themselves and via an autocrine loop, and paracrine signaling exerts pro-tumoral functions, so its serum concentration has been shown to correlate with tumor burden (37). IL-8 has been found to promote tumor growth, metastasis, chemo-resistance, and angiogenesis in different malignancies, including HNSCC (38–41). Serum levels of IL-8 were found to be consistently elevated in patients with recurrent or metastatic HNSCC (42).

To gain information about the predictive value of pre- and post-therapy cytokines in plasma and serum of HNSCC patients receiving ICB utilizing PD-1 antibodies for outcome, we set up a feasibility study and report about a signature linked to prolonged progression-free and OS.

## Materials and methods

### Study population and patient samples

The study was conducted according to the guidelines of the Declaration of Helsinki and approved by the Ethics Committee of the University Leipzig (vote NICEI-CIH 341-15-ff). Included in this study were patients with histopathologically confirmed HNSCC who received anti-PD-1 checkpoint inhibitor therapy between 2017 and 2022 at the university hospital of Leipzig in either curative or palliative settings. Subgroups of our cohort were participants of various studies including ADRISK (NCT03480672), NadiHN (EUDRA-CT 2016-004787-20), ELDORANDO (NCT03193931), CheckMate 651 (NCT02741570), and NIS-HANNA (NCT03114163).

Clinical data including TNM categories and staging according to criteria of Union for International Cancer Control (UICC) and information about the clinical course of patients were taken from the tumor database of the Otorhinolaryngology Department. Patient characteristics, such as their self-reported tobacco smoking history and status, and daily alcohol consumption and history, were collected at date of registration (**Table 1**).

**Table 1:** Baseline characteristics of the study population comparing patients receiving anti-PD-1 ICB in the curative or palliative setting.

		Total		Curative		Palliative				
		*N*	(%)	*n*	(%)	*n*	(%)	OR	(95% CI)	*p-*value^†^
Sex	Male	19	(76.0)	4	(57.1)	15	(83.3)	0.267	(0.038–1.862)	0.1686
	Female	6	(24.0)	3	(42.9)	3	(16.7)			
Age	<50 years	2	(8.0)	0	–	2	(11.1)			0.4838
	50–59 years	11	(44.0)	4	(57.1)	7	(38.9)			
	60–69 years	9	(36.0)	3	(42.9)	6	(33.3)			
	>70 years	3	(12.0)	0	–	3	(16.7)			
Body mass index (kg/m^2^)	BMI ≤25	17	(68.0)	4	(57.1)	13	(72.2)	0.513	(0.083–3.158)	0.4680
	BMI >25	8	(32.0)	3	(42.9)	5	(27.8)			
Reduced blood coagulation	No	14	(56.0)	5	(71.4)	9	(50.0)	2.500	(0.381–16.42)	0.3325
(anticoagulation therapy)	Yes	11	(44.0)	2	(28.6)	9	(50.0)			
Smoker status	Current	15	(60.0)	4	(57.1)	11	(61.1)	Ref.	(0.198–5.045)	0.9438
	Former	6	(24.0)	2	(28.6)	4	(22.2)	1.375	(0.178–10.65)	
	Never	4	(16.0)	1	(14.3)	3	(16.7)	0.917	(0.073–11.58)	
Tobacco smoking (pack years)	0 or <30	14	(56.0)	6	(85.7)	8	(44.4)	7.500	(0.753–75.72)	0.0620
	≥30	11	(44.0)	1	(14.3)	10	(55.6)			
Alcohol status	Current	15	(60.0)	5	(71.4)	10	(55.6)	Ref.	(0.219–4.564)	0.7365
	Former	4	(16.0)	1	(14.3)	3	(16.7)	0.667	(0.054–8.162)	
	Never	6	(24.0)	1	(14.3)	5	(27.8)	0.400	(0.036–4.411)	
Daily alcohol consumption	None	6	(24.0)	1	(14.3)	5	(27.8)			0.1144
(g/day)	1–30	6	(24.0)	4	(57.1)	2	(11.1)			
	31–60	8	(32.0)	1	(14.3)	7	(38.9)			
	>60	5	(20.0)	1	(14.3)	4	(22.2)			
Daily alcohol consumption	1–30	6	(24.0)	4	(57.1)	2	(11.1)	10.67	(1.309–86.94)	0.0155
(g/day)	Other	19	(76.0)	3	(42.9)	16	(88.9)			
Trial participation	ADRISK NadiHN ELDORANDO BMS NIS-HANNA NICEI-CIH	4 3 1 2 1 14	(16.0) (12.0) (4.0) (8.0) (4.0) (56.0)	4 3 0 0 0 0	(57.1) (42.9) – – – –	0 0 1 2 1 14	– – (5.6) (11.1) (5.6) (77.8)			<0.0001
T category Start ICB	T0	4	(16.0)	0	–	4	(22.2)			0.0025
	T1	6	(24.0)	2	(28.6)	4	(22.2)			
	T2	2	(8.0)	2	(28.6)	0	–			
	T3	3	(12.0)	3	(42.9)	0	–			
	T4	10	(40.0)	0	–	10	(55.6)			
N category Start ICB	N0	16	(64.0)	1	(14.3)	15	(83.3)			0.0033
	N1	3	(12.0)	3	(42.9)	0	–			
	N2b	3	(12.0)	2	(28.6)	1	(5.6)			
	N3b	3	(12.0)	1	(14.3)	2	(11.1)			
M category Start ICB	M0	18	(72.0)	7	(100)	11	(61.1)	9.783	(0.484–197.9)#	0.0518
	M1	7	(28.0)	0	–	7	(38.9)			
UICC 8th ed.	I	3	(12.0)	3	(42.9)	0	–			0.0112
	II	1	(4.0)	0	–	1	(5.6)			
	III	2	(8.0)	1	(14.3)	1	(5.6)			
	IVA	3	(12.0)	2	(28.6)	1	(5.6)			
	IVB	9	(36.0)	1	(14.3)	8	(44.4)			
	IVC	7	(28.0)	0	–	7	(38.9)			
p16 expression	No	13	(52.0)	3	(42.9)	10	(55.6)	Ref.	(0.161–6.201)	0.1874
	Yes	5	(20.0)	3	(42.9)	2	(11.1)	5.000	(0.551–45.39)	
	Missing	7	(28.0)	1	(14.3)	6	(33.3)	0.556	(0.047–6.629)	
Extracapsular extension	No ECE	4	(16.0)	3	(42.9)	1	(5.6)			0.0363
	ECE+	7	(28.0)	3	(42.9)	4	(22.2)			
	N0	7	(28.0)	1	(14.3)	6	(33.3)			
	Missing	7	(28.0)	0	–	7	(38.9)			
PD-L1 expression	CPS >1	16	(64.0)	4	(57.1)	12	(66.7)	Ref.	(0.202–4.955)	0.4458
	CPS <1	2	(8.0)	0	–	2	(11.1)	0.556	(0.022–13.93)#	
	Missing	7	(28.0)	3	(42.9)	4	(22.2)	2.250	(0.345–14.70)	
PD-L1 expression	CPS <20	11	(44.0)	1	(14.3)	10	(55.6)	Ref.	(0.055–18.30)	0.1752
	CPS >20	7	(28.0)	3	(42.9)	4	(22.2)	7.500	(0.59–95.38)	
	Missing	7	(28.0)	3	(42.9)	4	(22.2)	7.500	(0.59–95.38)	
Localization	LHSCC	7	(28.0)	1	(14.3)	6	(33.3)			0.4087
	OPSCC	10	(40.0)	4	(57.1)	6	(33.3)			
	OSCC	5	(20.0)	2	(28.6)	3	(16.7)			
	other	3	(12.0)	0	–	3	(16.7)			
Primary therapy	CRT	5	(20.0)	0	–	5	(27.8)			0.0918
	OP	5	(20.0)	0	–	5	(27.8)			
	OP+PORCT	7	(28.0)	4	(57.1)	3	(16.7)			
	OP+PORT	7	(28.0)	3	(42.9)	4	(22.2)			
	Pall. RT	1	(4.0)	0	–	1	(5.6)			
Context/reason for ICB therapy	Local R	8	(32.0)	0	–	8	(44.4)			0.0001
	Nodal R	1	(4.0)	0	–	1	(5.6)			
	Local + nodal R	2	(8.0)	0	–	2	(11.1)			
	M1	3	(12.0)	0	–	3	(16.7)			
	Local R +M1	4	(16.0)	0	–	4	(22.2)			
	Curative setting	7	(28.0)	7	(100)	0	–			
Reason for palliative therapy—	None	10	(40.0)	7	(100)	3	(16.7)	66.43	(3.026–1458.2)	0.0001
local/locoregional inoperable	Yes	15	(60.0)	0	–	15	(83.3)			
Reason for palliative therapy—	None	18	(72.0)	7	(100)	11	(61.1)	9.783	(0.484–197.86)	0.0518
M1 inoperable	Yes	7	(28.0)	0	–	7	(38.9)			
12 months ICB completed	No	20	(80.0)	4	(57.1)	16	(88.9)	0.167	(0.02–1.358)	0.0748
	Yes	5	(20.0)	3	(42.9)	2	(11.1)			

Curative, treated in ADRISK (NCT03480672) or NadiHN (EudraCT 2016-004787-20). Palliative, treated in palliative trials; n (%), number and percentage of patients. OR, odds ratio; 95% CI, 95% confidence interval. ^†^p-value from **χ^2^** tests with Bonferroni correction; Ref., reference category defined as equaling 1. ^#^Odds ratio calculated according to Cox and Haldane by adding 0.5 to each cell to prevent division by zero caused by empty cells. CPS, combined positive score. LHSCC, laryngeal (ICD-10-C32) and hypopharyngeal (ICD-10-C13) squamous cell carcinoma (SCC). OPSCC, oropharyngeal SCC (ICD-10-C01, C05, C09, C10). OSCC, oral SCC (ICD-10-C02, C04, C06); other includes one patient each with SCC of unknown primary (ICD-10-C77), sinus maxillaris (ICD-10-C31), and nasal cavity (ICD-10-C30). CRT, concurrent chemo-radiotherapy; OP, surgical resection; OP+PORCT, surgical resection followed by post-operative concurrent chemo-radiotherapy in ADRISK; OP+PORT, surgical resection followed by post-operative radiotherapy in NadiHN; pall. RT, palliative radiotherapy; local R, (inoperable) local recurrence; nodal R, (inoperable) nodal recurrence; local + nodal R, local and nodal recurrence; M1, distant metastasis; local R + M1, local recurrence and distant metastasis.

### Materials

We used serum-gel and EDTA-plasma S-Monovettes^®^ (Sarstedt, Nübrecht, Germany) to collect venous blood samples from patients according to standardized operating procedures. Approximately 60 min after blood draw, Monovettes were centrifuged for 10 min at 2,720 x ***g***. Aliquoted serum and plasma samples were stored at −80°C until analysis.

To measure the cytokine concentrations in serum and plasma, we performed indirect *Sandwich* ELISAs. OptEIA™ Kits (BD Biosciences, Heidelberg, Germany) were utilized to quantify IL-6, IL-8, IP-10, MCP-1, and IFN-γ, and VEGF-EDK kits (#900-K10; Peprotech, Hamburg, Germany) were utilized to quantify VEGF according to the manufacturers’ instructions. Dulbecco’s phosphate-buffered saline (PBS) from Biochrom AG (Berlin, Germany) was used for coating the microtiter plates (Greiner Bio-One, Nürtingen, Germany) with 50 μl/well diluted capture antibody overnight at 4°C. PBS containing 0.025% Tween^®^ 20 from Sigma-Aldrich (Darmstadt, Germany) was used for washing. After a 30-min blocking step with PBS containing 5% heat-inactivated fetal calf serum (FCS; Thermo-Fisher Scientific, Waltham, MA, USA), 50 μl of sample (plasma or serum) and a serial dilution of the appropriate standard for calibration were incubated for 120 min, followed by washing and adding 50 μl/well biotinylated antibodies. After three further washing steps, streptavidin-horseradish peroxidase conjugate (HRP) was added to the wells and incubated for 60 min followed by six washing steps. Then tetramethyl benzidine 1-Step^TM^ Ultra (Pierce via Thermo-Fisher Scientific, Waltham, MA, USA) was added as the substrate. TMB 1-Step™ Ultra conversion by HRP was stopped by adding the same volume of 1 M sulfuric acid. After measuring optical densities at λ_1 _= 450 nm and λ_2 _= 620 nm on the Synergy2™ multi-mode microplate reader (BioTek Instruments, Inc., Winooski, VT, USA), we calculated the calibration curves using Gen5™ software (BioTek Instruments, Inc., Winooski, VT, USA). We converted optical densities to pg/ml concentrations using four-parameter calibration curves. The lower limit of detection and the lower limit of quantification were calculated as described and were always ≤ 4 pg/ml for all cytokines.

To obtain a unique measure, mean values of at least one pre-therapy sample and mean of at least two measurements from samples taken at a minimum of 2 weeks after first cycle ICB were used. We compared cytokine concentrations in serum or plasma according to various reference points with or without normalization to estimate changes in cytokine levels related to ICB. We used the derived values of the individual patient to identify in receiver-operating characteristic (ROC) curves the optimum cut-off values (maximum Youden index) for binary classification patients to assess their impact on OS.

### Statistical analysis

Patient characteristics and follow-up data were analyzed in relation to the results from ELISA measurements and categorization according to ROC curves as described above. We also analyzed clinical characteristics of patients, and lifestyle-associated risk factors (daily alcohol consumption categorized in 0, 1–30 g, 31–60 g, and >60 g) and status (never, former, and current), tobacco smoking (total number of pack years smoked during lifetime), and smoking status (never, former, and current). Clinical characteristics of patients included age; sex; T, N, and M categories; HPV status (according to p16 immune histochemistry); and treatment modalities (curative vs. palliative setting; ICB with vs. without chemotherapy; pembrolizumab vs. nivolumab). Associations between categorical variables were examined by Pearson’s chi-square test. We calculated overall survival (OS) time from date of first cycle ICB to date of death (event) or end of follow-up (censored) and tumor-specific survival (TSS) time from date of first cycle ICB to date of cancer-related death (event) censoring other causes of death or end of follow-up.

We analyzed survival using the Kaplan–Meier method (43) applying log-rank tests (44) and hazard ratios (HR) using Cox proportional hazard models (45) utilizing the conditional logistic regression forward method, and bootstrapping (46) (SPSS version 27, IBM Corporation, Armonk, New York). We considered two-sided *p <*0.05 as significant.

## Results

Of the 45 patients registered in RCT utilizing anti-PD-1 ICB, 25 patients were randomized to receive per protocol doses according to the respective study protocol. The characteristics of patients are shown in **Table 1**.

**Figure 1:**
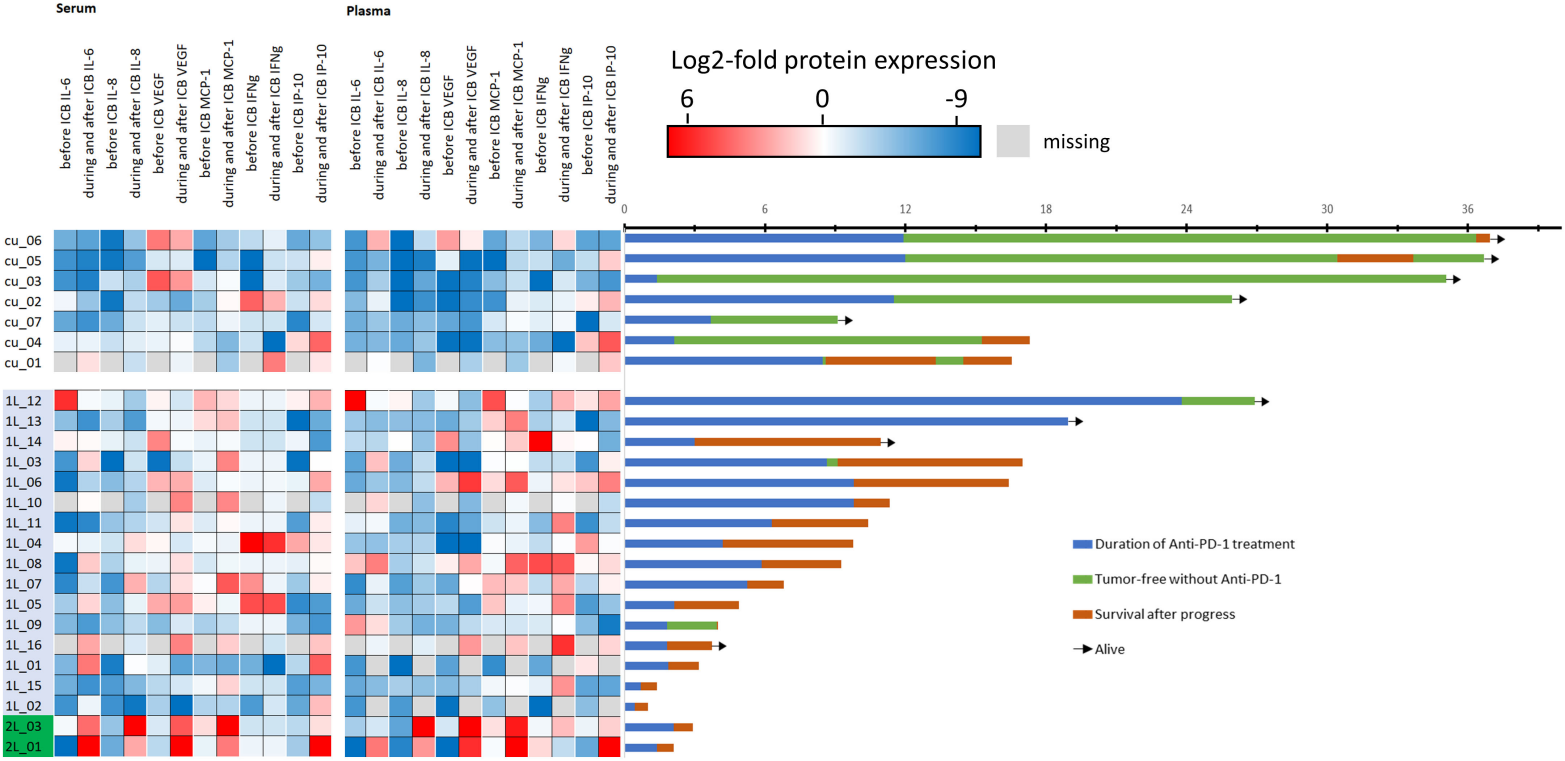
Heatmap showing each patient’s cytokine expression level after normalization to mean of all 50 values for the particular cytokine pre- (before) and post-ICB (during/after start of ICB). Blue denotes low, red denotes high level of the particular cytokine (please note log2 scaling) and swimmer plots showing the individual course of 25 patients. The first seven patients (cu_01 to cu_07) are treated in the curative setting, whereas 1L denotes 16 patients in the first-line and 2L denotes two patients treated in the second line setting. The timeline of the swimmer plots is from the start of immune-checkpoint blockade (ICB) utilizing anti-PD-1 antibodies with the day of first cycle, the duration of treatment until progressing disease (PD), side effects including immune-related adverse events, or end per protocol, followed by either disease-free survival or survival after PD until end of follow-up. Arrows indicate patients alive.

The outcome of patients in the curative setting was superior as shown in **Figure 1** (right panel, swimmer plots).

However, some R/M HNSCC patients also had good outcome and were alive at end of follow-up. A total of 54 pre- and 101 post-ICB serum and 54 pre- and 94 post-ICB plasma samples were available for measurement. The mean concentration of all cytokines with the respective confidence interval is shown in **Table 2**.

**Table 2:** Mean and (in brackets) 95% CI of concentrations of cytokines indicated in serum and plasma of 25 HNSCC patients before (pre-ICB) and after start of immune-checkpoint inhibitor therapy (post-ICB).

	Serum (pg/ml)		Plasma (pg/ml)	
	pre-ICB	post-ICB	pre-ICB	post-ICB
IFN-γ	12.99 (7.49–18.48)	9.22 (5.77–12.67)	26.07 (9.83–42.31)	50.83 (32.69–68.97)
VEGF	249.5 (196.2–302.8)	320.1 (243.8–396.4)	25.97 (13.96–37.98)	50.5 (17.95–83.05)
MCP-1	409.48 (341.2–477.8)	588.7 (450.6–726.8)	202.6 (159.8–245.4)	251.84 (199.7–304.0)
IL-8	15.73 (9.51–21.94)	196.7 (0.00–481.1)	14.11 (7.34–20.89)	159.27 (0.00–415.2)
IL-6	17.56 (0.00–37.11)	25.56 (6.37–44.74)	126.95 (0.00–284.9)	62.14 (33.47–90.8)
IP-10	219.5 (163.0–276.0)	553.2 (294.9–811.5)	504.44 (394.4–614.5)	829.78 (537.7–1121.8)

As shown in **Table 2**, the concentration in serum and plasma differed significantly in post-ICB IFN-γ, pre- and post-ICB VEGF, pre- and post-ICB MCP-1, and post-ICB IL-6 and pre-ICB IP-10. The pre- and post-ICB serum concentration differed significantly for MCP-1, and the pre- and post-ICB plasma concentration differed significantly for IP-10. The corresponding *p*-values for patients–individual comparisons according to the *t*-test for paired samples are shown in **Table 3**.

**Table 3:** *p-*values from two-sided paired *t*-tests comparing concentrations in EDTA-anticoagulated blood and serum of HNSCC patients before (pre-ICB) and after start of immune-checkpoint inhibitor therapy (post-ICB) for cytokines indicated.

Comparison	IFN-γ	VEGF	MCP-1	IL-8	IL-6	IP-10
Pre-ICB vs. post-ICB serum	0.2677	0.1499	**0.0305**	0.2308	0.5753	**0.0210**
Pre-ICB vs. post-ICB plasma	0.0549	0.1828	0.1654	0.2855	0.4406	0.0530
Pre-ICB plasma vs. serum	0.1480	**<0.0001**	**<0.0001**	0.7357	0.1929	**<0.0001**
Post-ICB plasma vs. serum	**0.0002**	**<0.0001**	**<0.0001**	0.8515	**0.0471**	0.1780

Significant *p*-values are in bold.

As we noticed different ICB-related changes, in particular cytokine levels (**Figure 1**, left panel), we used ROC analyses to define optimum cutoff values for the particular cytokine and sample type according to OS. The results are shown in **Table 4**.

**Table 4:** Results of receiver operating characteristic (ROC) analyses of various measures of cytokines in serum or plasma of HNSCC undergoing PD-1 ICB and overall survival.

	Cut-off	Sensitivity	FDR	Specificity	Youden index	AUC	(95% CI)	*p-*value^‖^
IL-6 (pg/ml) post-ICB (serum)	6	0.786	0.250	0.750	0.589	0.777	(0.575–0.979)	**0.034**
IL-6 (pg/ml) post–pre-ICB (serum)	−0.12	0.750	0.000	1.000	0.750	0.844	(0.666–1.000)	**0.011**
IL-8 (pg/ml) post-ICB (serum)	34	0.643	0.000	1.000	0.643	0.795	(0.605–0.984)	**0.024**
IL-8 (pg/ml) post–pre-ICB (serum)	30	0.583	0.000	1.000	0.583	0.854	(0.687–1.000)	**0.009**
IP-10 delta pre–post-ICB (% pre; serum)	10	0.643	0.125	0.875	0.563	0.679	(0.449–0.908)	0.172
VEGF (pg/ml) post–pre-ICB (serum)	20	0.833	0.000	1.000	0.833	0.938	(0.828–1.000)	**0.001**
VEGF post in % pre (serum)	100	0.786	0.125	0.875	0.688	0.795	(0.597–0.992)	**0.024**
IL-8 (pg/ml) post-ICB (plasma)	12	0.750	0.250	0.750	0.563	0.781	(0.568–0.994)	**0.037**
IFN-γ (pg/ml) pre-ICB (plasma)	18	0.583	0.125	0.875	0.510	0.667	(0.408–0.926)	0.217
IFN-γ (pg/ml) post-ICB (plasma)	30	0.750	0.375	0.625	0.469	0.677	(0.438–0.916)	0.190
MCP-1 delta pre–post-ICB (% pre; plasma)	15	0.875	0.357	0.643	0.563	0.768	(0.546–0.990)	**0.041**

FDR, false discovery rate; AUC, area under the (ROC) curve. ‖, two-sided p-value; IL-6, interleukin 6; post–pre-ICB = difference post–pre (in); IL-8, interleukin 8 aka CXCL8. IP-10, interferon-induced protein 10 aka CXCL10; delta pre–post = (post/pre)-1; post in % pre = post/pre, given in percent. VEGF, vascular endothelial growth factor A; IFN-γ, interferon gamma; MCP-1, monocyte chemoattractant protein 1 aka CCL2.

Significant p-values are in bold.

**Figure 2:**
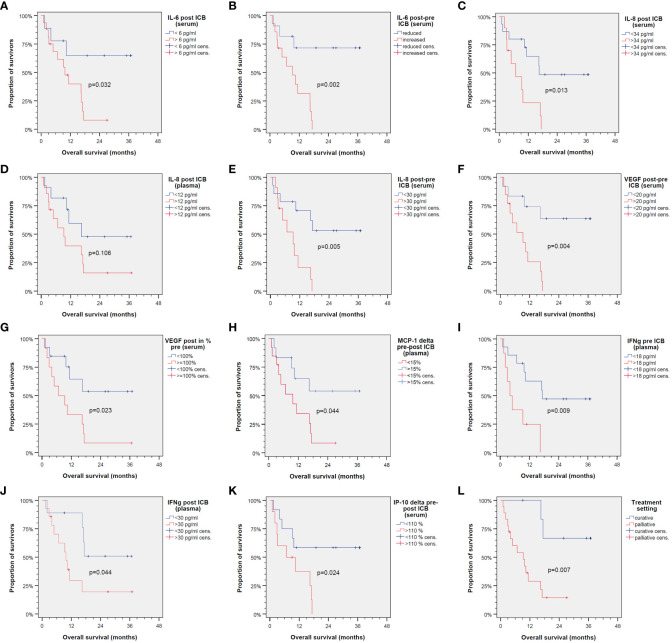
Kaplan–Meier cumulative survival plots for overall survival of 25 patients treated with immune-checkpoint blockade (ICB) utilizing anti-PD-1 antibodies. Binary split of the cohort according to optimum cutoff values for cytokine expression patterns shown in **Table 2** with the only exception of IL-8 in plasma post-ICB **(D)** showing significant different overall survival between groups **(A–C, E–K)** as is the same comparing ICB in the curative vs. palliative setting **(L)**. *p*-values shown are from log-rank tests (two-sided).

Using the cutoffs for binary split of the cohort, we used Kaplan–Meier cumulative survival plots to identify significant differences in OS (**Figure 2**).

**Figure 3:**
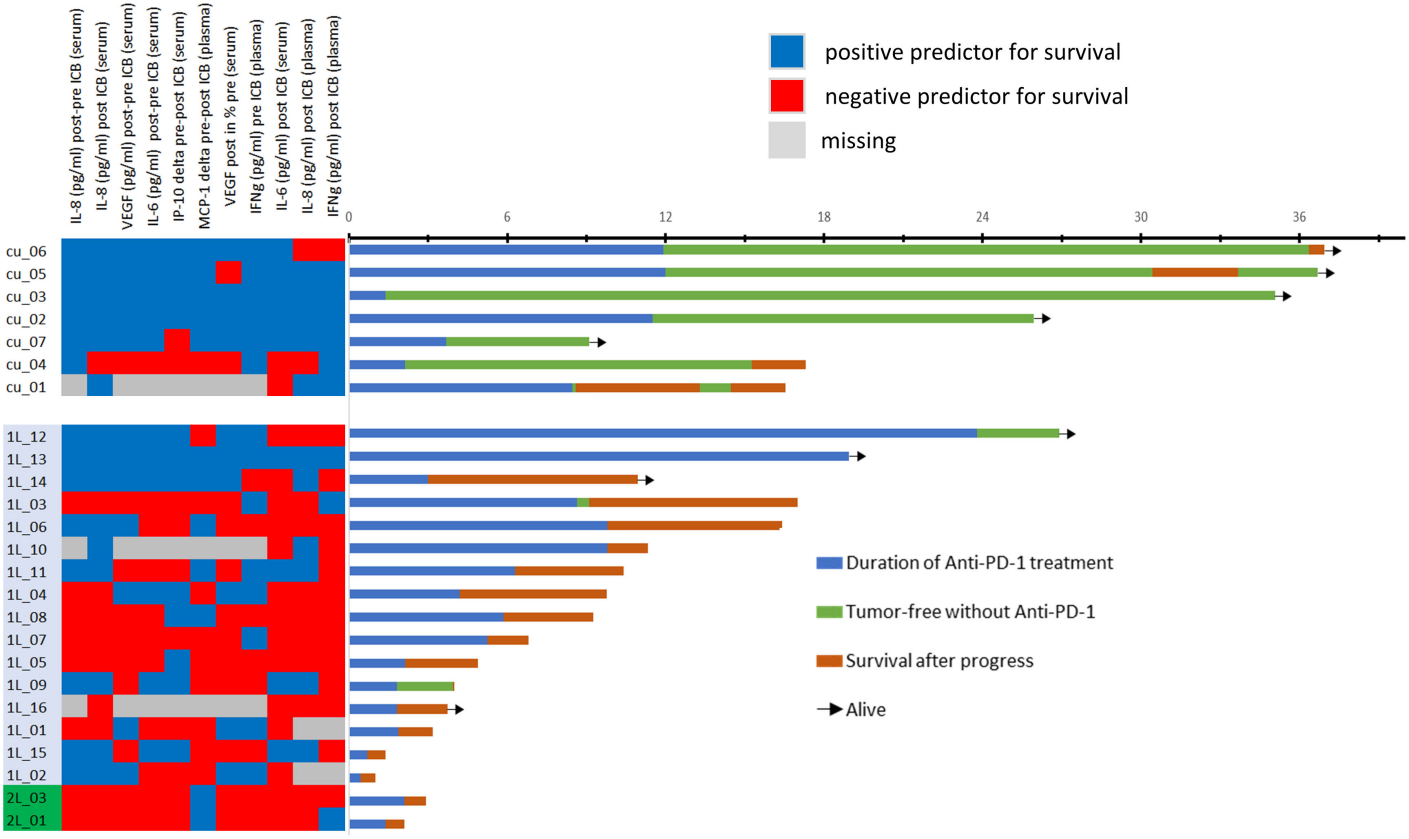
Heatmap showing the categorization of each patient’s cytokine expression linked to rather favorable (blue) or poor outcome (red) according to optimum cutoff values shown in **Table 2** (left) and swimmer plots showing the individual course of 25 patients. Explanation of swimmer plots same as in **Figure 1**.

However, due to significant correlation of particular cytokines and overlap of cytokine expression patterns among patients (**Figure 3**), some OS curves suggest similar impact of particular cytokines on outcome after ICB.

**Figure 3** shows a heatmap for all cytokines measured in the particular sample type linked to deviating outcome according to significant different OS. Interestingly, some cytokines show close correlation, and cytokine expression levels accompanied by either improved (blue) or impaired OS (red) cluster together. To identify the most important predictors of OS among cytokines, we used Cox proportional hazard regression modeling applying the stepwise forward method and identified five cytokines and growth factors being independent predictors of OS (**Table 5**).

**Table 5:** Independent predictors of overall survival (OS) of HNSCC undergoing PD-1 ICB identified in the multivariate Cox proportional hazard regression model automatically build applying the step-wise forward likelihood ratio method.

**Covariate**	**Ref.**	**Characteristic**	***n***	**OS events** ***n* (%)**	***p*-value ^#^**	**Cox univariate HR (95% CI)**	***p-*value^†^**	**Cox multivariate HR (95% CI)**	***p-*value^††^**	**Loss^‡^ in *χ* ^2^**	***p-*value^‡^**	***p-*value^‡‡^**
VEGF post–pre-ICB (serum)	< 20	≥ 20	10	10 (100)	**0.004**	5.634 (1.722–18.437)	**0.0043**	4.179 (0.908–19.241)	0.0664	3.565	0.059	0.178
IP-10 delta pre–post-ICB (serum)	< 10%	≥ 10%	10	9 (90.0)	**0.024**	3.559 (1.167–10.855)	**0.0257**	5.322 (1.293–21.905)	**0.0206**	5.720	**0.017**	**0.036**
MCP-1 delta pre–post-ICB (plasma)	≥ 15%	< 15%	10	9 (90.0)	**0.044**	3.142 (1.046–9.432)	**0.0413**	4.852 (0.968–24.309)	0.0547	4.451	**0.035**	**0.041**
IFN-γ pre-ICB (plasma)	< 18	≥ 18	8	7 (87.5)	**0.009**	5.414 (1.587–18.474)	**0.0070**	6.544 (1.462–29.287)	**0.0140**	6.597	**0.010**	**0.012**
IFN-γ post-ICB (plasma)	< 30	≥ 30	14	11 (78.6)	**0.044**	4.441 (1.191–16.563)	**0.0264**	12.105 (1.604–91.332)	**0.0156**	6.847	**0.009**	**0.048**

Shown are number (n) of patients with the respective characteristic accompanied by the number (n) and percentage (%) of deaths observed and the p-values from log-rank tests, univariate and multivariate Cox proportional hazard regression models as indicated.

^#^p-value from log-rank tests applied to Kaplan–Meier cumulative survival plots; ^†^p-value from univariate Cox proportional hazard regression analysis. ^††^p-value from multivariate Cox proportional hazard regression analysis; ^‡^ loss in **χ^2^** by excluding the covariate from the multivariate Cox proportional hazard regression model and the respective p-value. ^‡‡^p-value from multivariate Cox proportional hazard regression analysis applying the bootstrap using 1,000 iterations. All p-values shown are two-sided; significant p-values in bold.

**Figure 4:**
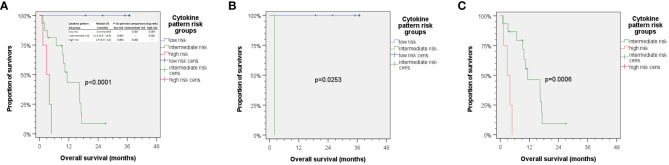
Kaplan–Meier cumulative survival plots for overall survival of 25 HNSCC treated with ICB. **(A)** Aggregating the five independent predictors for OS via summarizing the natural logarithm of their hazard ratio into a cytokine expression pattern (CEP) score allows for calculating individual risk for cancer-related death, so patients can be categorized as being CEP^low^, CEP^int^, or CEP^high^ accordingly correlating with rather low, intermediate, or high risk of death. The insert in panel **(A)** shows median overall survival in risk groups with *p*-values from pairwise comparison of CEP groups indicated. **(B)** HNSCC patients with <30 pack years and 1–30 g/day predominantly were CEP^low^ with missing CEP^high^, while those in **(C)** had higher alcohol exposure and >30 pack years and were either CEP^int^ or CEP^high^ with missing CEP^low^. *p*-values shown are from two-sided log-rank tests.

We also included clinical parameters in the model, and in particular those with *p* < 0.2 in univariate analyses (> 30 pack years; > 30 g/day alcohol consumption). However, no clinical parameter was found to be an independent predictor for OS when added to the multivariate Cox proportional hazard model with the five cytokines VEGF (post–pre-ICB; serum), IP-10 (delta pre–post-ICB; serum), MCP-1 (delta pre–post-ICB; plasma), IFN-γ (pre-ICB; plasma), and IFN-γ (post-ICB; plasma). Using the natural logarithm of the hazard ratio according to the cutoff values for binary split of these five cytokines (**Tables 4**, **5**) and calculating the sum of the cytokine expression pattern (CEP)-associated hazard, we were able to categorize each of the patient’s probability for dying into the three groups with either low (CEP^low^), intermediate (CEP^int^), or high risk (CEP^high^). Overall survival differed significantly between these risk groups. The respective outcome is shown in **Figure 4**.

While we did not observe any correlation between either mono-ICB or ICB +platinum or ICB in curative or palliative setting when added to the Cox model of five CEP defining cytokine measures, a strong correlation between lifestyle-associated (and therefore modifiable risk factors) tobacco smoking history and daily alcohol consumption was observed. Stratification of the cohort according to lifestyle-related risk factors tobacco smoking and daily alcohol consumption with (B) rather low and (C) rather high exposure to lifestyle-related risk factors demonstrates the correlation of cytokine patterns with lifestyle-related risk groups (**Figure 4**; compare B, <30 pack years and 1–30 g/day, and C, >30 pack years and higher alcohol exposure). Favorable outcome was observed in patients with low to moderate alcohol consumption and below 30 pack years, as they had low risk for death according to cytokine patterns. These patients responded to ICB in a favorable way, and none of them died. The risk profile according to smoking and drinking correlated significantly with the cytokine pattern risk group (*r* = 0.745) and hence explained 55.5% variance regarding this classification. In line with these findings, it might be of particular interest that multivariate Cox regression without inclusion of *Pi* among the measured cytokines only extracted alcohol consumption and smoking as independent predictors of OS in this small cohort, and neither mono-ICB or ICB plus platinum nor applying ICB in curative or palliative setting emerged as significant independent predictors of OS.

## Discussion

Adaptive immune resistance is the summary of all mechanisms that a tumor cell uses to adapt to the changes in the TIME and eventually overcome immune attack (47). Acquired mutations and loss of transcriptional control due to hypo-methylation of gene-promotor regions as a result of the selective pressure caused by cell proliferation result in many pathways including cytokine signaling pathways involving the synthesis of cytokines, which create a favorable TIME for escaping immune-mediated destruction (48).

We identified four cytokines as being the only independent predictors of OS among our patients. These four cytokines are VEGF (difference between pre- and post-ICB serum), IP-10 (change between pre- and post-ICB serum), MCP-1 (change between pre- and post-ICB plasma), and IFN-γ (quantity in pre- or post-ICB plasma).

The interferons are divided into type I and type II according to receptor specificity and sequence homology, with IFN-γ being the sole type II interferon. IFN-γ is the immune interferon and produced by a variety of cells, including CD4+ and CD8+ lymphocytes, NK cells, and B cells. Tumor-infiltrating lymphocytes (TILS) are the main source of IFN-γ in the TIME. IFN-γ exerts many anti-tumoral functions after binding to the IFN-γ receptor. The downstream target genes of IFN-γ, besides modulating innate and adaptive immune responses, are related to the regulation of cell cycle, apoptosis, and inflammation (49). IFN-γ has been shown to decrease tumor cell growth by enhancing expression of cell cycle inhibitor proteins p27Kip, p16, or p21 in various cancer types (50, 51). Another important function of IFN-γ is the regulation of cell-surface class I and II MHC expression (49). In tumor cells, IFN-γ upregulates the expression of MHC class I molecules; their increased expression is related to enhanced antigenicity of the cell due to increased presentation of peptides including those derived from tumor-associated antigens (52). Besides acting on the tumor cell itself, IFN-γ can also act on the tumor stroma. IFN-γ signaling on endothelial cells leads to blood vessel regression in the tumor and, therefore, an arrest of blood flow (53). In addition, IFN-γ signaling causes tumor infiltration macrophages (TAMs) to differentiate to M1 macrophages, which suppress VEGF secretion and thereby inhibit angiogenesis (54).

Despite the central antitumoral role of IFN-γ during initiation of an immune response, it can also exert pro-tumoral functions (55). Exposure to elevated IFN-γ levels and a prolonged exposure, in particular, exert selective immune pressure on the tumor cell, which leads to a loss of genes involved in antigen presentation, such as MHC class I (56). Moreover, IFN-γ is able to induce gene-expression patterns linked to multigenic resistance (57). Myeloid-derived suppressor cells (MDSCs) are myeloid-origin cells, which are induced by tumor- and host-secreted factors and are present in most cancer patients. MDSC can suppress T-cell activation and therefore downregulate immune surveillance and antitumor immunity. The development and function of most MDSC requires IFN-γ (58). IFN-γ produced by CD8^+^ T cells can trigger the expression of induced nitric oxide synthase (iNOS) in certain MDSC, which contributes to the immunosuppressive activity of these MSDC (59). Prior studies in HNSCC also indicate that the IFN-γ-induced nitric oxide synthase supports tumor progression and lymphatic spread in HNSCC (60). Previous studies show elevated levels of IFN-γ (3.86 ± 10.07 pg/ml) in serum of HNSCC patients compared to healthy individuals (61). Median pre- and post-treatment IFN-γ serum levels (33.5 pg/ml pre-treatment versus 28.8 pg/ml post-treatment) were non-significant for newly presenting HNSCC patients (62). IFN-γ levels in plasma have been rarely assessed in HNSCC patients. Our results show that higher pre- and post-therapeutic levels of IFN-γ (>16 pg/ml pre-ICB and >30pg/ml post-ICB in plasma) are negative predictors for response to anti-PD-1 checkpoint inhibitor therapy and negative predictors for overall survival. IFN-γ induces PD-L1 expression (2). The selective induction of PD-L1 by the tumor cell is the first clearly defined and therapeutically validated mechanism of adaptive immune resistance (47). During the upregulation of PD-L1 by IFN-γ, the tumor utilizes IFN-γ as part of a negative feedback loop that inhibits anti-tumoral immune responses (63, 64). Persistent IFN-γ signaling also allows the tumor to acquire signal transducer and activator of transcription 1 (STAT1)-related epigenomic changes and augments expression of interferon-stimulated genes and ligands for multiple T-cell inhibitory receptors, which can be seen as a mechanism of adaptive resistance to checkpoint inhibitor therapy. Biomarkers for interferon-driven resistance are reported as being associated with clinical progression after anti-PD-1 therapy (57). This is consistent with our findings. Another mechanism contributing to the development of adaptive resistance to anti-PD-1 immunotherapy is the activation of the tumor-intrinsic NOD-, LRR- and pyrin domain-containing protein-3 (NLRP3) inflammasome–heat shock protein 70 (HSP70) signaling axis. The NLRP3–HSP70 axis recruits granulocytic polymorph-nuclear myeloid-derived suppressor cells (PMN-MDSCs; see above and (56, 58, 59)) into the tumor microenvironment, which are major regulators of tumor immune suppression and support disease hyperprogression in response to anti-PD-1 immunotherapy. This signaling axis is triggered by CD8^+^ T-cell cytotoxicity and is enhanced by IFN-γ (65). It has been sown that the IFN-γ-related mRNA profile predicts the clinical response to PD-1 blockade (66, 67).

Monocyte chemoattractant protein-1 (MCP-1/CCL2) is a member of the C–C chemokine family and a potent chemotactic factor. It is primarily produced by monocytes and macrophages and regulates the migration and infiltration of monocytes, T cells, and natural killer cells (68). MCP-1 is produced by many cancer cells and acts on the very same by signaling through C–C chemokine receptor type 2 (CCR-2, CD192) on CCR-2-expressing cancer cells. This encourages tumor growth and invasiveness. Furthermore, MCP-1 triggers angiogenesis and tumor development by either recruiting monocytes into the TIME, which differentiate into tumor-associated macrophages (TAMs) or acting directly on endothelial cells to produce endothelial growth factors (48). Several studies found MCP-1 to be a negative prognostic factor in HNSCC, as high levels of MCP-1 in the TIME lead to a poor prognosis and impaired outcome (69, 70), but there is not much data on serum and/or plasma levels of MCP-1 in HNSCC patients available in the literature. One previous study found no significant difference in mean pre-therapeutic MCP-1 serum levels in HNSCC patients compared to healthy controls (45.27 ± 16.43 pg/ml versus 60.09 ± 21.83 pg/ml) (71). On the other hand, high serum levels of MCP-1 correlated with favorable outcome in breast (>250 pg/ml) and pancreatic cancer (>91 pg/ml) patients (72, 73). This is consistent with our results, as we also found a correlation of higher MCP-1 levels, albeit in plasma, and improved OS. This opposite effect of MCP-1 has been investigated by our group before (74). MCP-1-associated increased recruitment of monocytes into the TIME eventually leads to their differentiation into TAMs. These TAMs can either be M1 and have tumoricidal capacity or M2 and promote tumor progression by inducing vascularization and tumor growth (75, 76). Indeed, it was shown that monocytes (the macrophage precursor cells) entering the tumor can differentiate into these two categories, M1 classical activated macrophages, for example under the stimulation of IFN-γ, and M2 alternative activated macrophages. Type M1 macrophages secrete pro-inflammatory cytokines and present tumor-specific antigens through expression of the MHC classes I and II. M1 macrophages therefore promote antitumor immunity, whereas M2 macrophages exert tumor-promoting activities (77).

Interleukin 8 (IL-8, CXCL8) is a pro-inflammatory cytokine that was first named neutrophil-activating factor (NAF) due to its ability to stimulate neutrophil exocytosis and oxidative burst (78). Different cell types including monocytes, macrophages, fibroblasts, endothelial cells, and epithelial cells secrete IL-8. Various cytokines (e.g., IL-6 and TNF-α) and environmental stresses such as hypoxia, reactive oxygen species, and bacterial particles (79) stimulate its expression. Downstream signaling of IL-8 is mediated through extracellular binding to either of two G-protein-coupled receptors, C-X-C chemokine receptor type 1 (CXCR1), and type 2 (CXCR2), which are expressed on monocytes, granulocytes, and endothelial cells (80). The activation of CXCR1/2 results in calcium mobilization from the endoplasmic reticulum and the activation of protein kinase C (PKC), which is critical for neutrophil chemotaxis (81). Activation of one of the two receptors also induces granule release in neutrophils, and CXCR1 induces superoxide anion production, which is essential for the IL-8-mediated oxidative burst (82).

Interleukin-8 has been found to promote tumor progression by altering the TIME in favor of the tumor promoting angiogenesis (83). One mechanism used by cancer cells to acquire motility and invasiveness is the epithelial-to-mesenchymal transition (EMT), which involves loss of epithelial cell-to-cell contacts and increased expression of mesenchymal proteins that mediate motility (e.g., fibronectin) (84). IL-8 has been found to promote this transition, resulting in increased tumor cell migration and development of metastases (85). Increased levels of IL-8 have been reported in various cancer types and are associated with late-stage disease and reduced overall survival (86–88). IL-8 signaling through CXCR2 recruits MDSCs to the tumor side, which, as stated above, can cause resistance to anti-PD1 therapy by inhibiting T-cell infiltration and activation (89). In melanoma and NSCLC patients treated with PD-1 inhibitor therapy, serum levels of IL-8 decreased at the time of best response and serum levels rose in non-responders at the time of disease progression (90). This is consistent with our results, as we found that higher serum and plasma levels after immune checkpoint blockade (ICB) correlated with impaired overall survival.

IL-8 also induces chemoresistance in tumor cells by upregulating the ATP-binding cassette subfamily B member 1 (ABCB1), which leads to the production of multidrug resistance protein 1 (MDR1), a protein linked with drug resistance (91, 92). This mechanism was found to mediate chemoresistance to cisplatin in gastric cancer as high pre-therapeutic serums levels of IL-8 (446.71 ± 111.07 pg/ml) predicted poor response to platinum-based chemotherapy (93). In HNSCC, IL-8 serum levels are elevated (123.47 ± 282.66 pg/ml) compared to healthy controls and are correlated with loco-regional metastases (61). This is consistent with our findings, as higher post-ICB serum and plasma levels correlated with impaired OS.

IP-10 (CXCL10) is an IFN-γ-induced protein belonging to the CXC chemokine family, which is able to reduce tumor growth, regulate angiogenesis, and increase the recruitment of cytolytic lymphocytes into tumor lesions (94). The chemokines IL-8 and IP-10 (CXCL10) have been identified as biomarkers that improved the prediction of lung cancer incidence in combination with lung cancer risk models (95). Mean pre-therapeutic IP-10 serum levels in HNSCC patients were found to be significantly elevated compared to healthy controls (2,502.8 ± 1,098.5 pg/ml versus 1,488.3 ± 510.4 pg/ml) (71). In our previous work, we already established an IP-10 score that was based on *ex-vivo* response of HNSCC to pembrolizumab predicted treatment outcomes in HNSCC patients (28).

IL-6 is a well-known pleiotropic cytokine involved in pro-inflammatory immune responses, autoimmune diseases, senescence, and carcinogenesis. Signaling via its receptor IL-6R-α linked to gp130 activates phosphorylation of the signal transducer STAT3, and increased STAT3 signaling through elevated IL-6 was associated with reduced overall survival in p16-negative HNSCC (96). The activation of the STAT3 pathway was also found to promote PD-1/PD-L1 expression and therefore might play an important role in the antitumor immune response of HNSCC (97). HNSCC patients show significantly higher mean IL-6 serum levels compared to healthy controls (19.5 pg/ml versus 6.0 pg/ml (29); 14.1 pg/ml versus 9.1 pg/ml (71)). Higher IL-6 levels are correlated with higher tumor stage and disease-positive lymph nodes in HNSCC patients (29).

A significant decrease in serum IL-6 and IL-8 levels between pre- and post-treatment samples was observed in newly presenting HNSCC patients after initial treatment (62), which suggests that decreasing levels of IL-6 and IL-8 may function as good prognostic factors under pembrolizumab treatment as well. Increased levels of IL-8 and IFN-γ were correlated with loco-regional metastases in patients with laryngo-pharyngeal HPV-positive cancers (61).

Mean VEGF serum levels are significantly higher in HNSCC patients than in healthy individuals (144.5 pg/ml versus 32.7 pg/ml) (98). Lower pre-therapeutic VEGF plasma levels (<26 pg/ml) were found to be a positive prognostic biomarker for prolonged progression-free survival in HNSCC patients (27). The interplay of cancer cells and immune cells in the TIME of HNSCC consequently appears to be mirrored by cytokines and growth factors including VEGF (27) that could be measured in serum and plasma representing valuable liquid biopsies. Our observation that not each and every biomarker can be reliably measured in different blood sample types, serum, or plasma and used to gain information about the particular HNSCC and the prognosis of the patient or to predict response to treatment is not new (27). Despite being drawn at the same point of time, serum and plasma undergo changes related to blood clotting or its prevention through anticoagulants, and this obviously is reflected by the superiority of either serum or plasma for developing biomarkers to predict outcome. This also applies to outcome after ICB. Despite the close correlation of a number of cytokines measured in serum and plasma, we found five covariates derived from cytokine measurements being independent predictors for OS. Three of them require calculation of differences between pre- and post-ICB initiation samples, while IFN-γ concentrations in plasma pre- and post-ICB alone are sufficient independent predictors in the model. The three CEP-risk groups had different risk for dying, as the groups had median (95% confidence interval) OS of 2.9 (0.4–5.4) and 11.3 (8.8–13.8) months in CEP^high^ and CEP^int^ groups and no death within mean follow-up of 29.2 (22.1–36.2) months. Of particular interest, however, is the close link between the cytokine pattern risk group and lifestyle-associated risk factors smoking and alcohol consumption (*r* = 0.745), which explained 55.5% variance regarding outcome after ICB.

Besides some strengths according to standardized blood draw and handling of serum and plasma and SOPs for cytokine measurements, our study and the transferability of findings have numerous limitations. The most important limitation is the small number of patients and the heterogeneity of treatment, which probably have confounded the outcome of patients studied. However, our sensitivity analyses and multivariable analyses applying Cox regression and internal validation by bootstrapping revealed no substantial impact on CEP if ICB was applied either alone or combined with cisplatin-based chemotherapy or used as adjuvant treatment after surgery of LA-HNSCC or first-line therapy of R/M HNSCC. However, the close correlation of lifestyle-associated risk factors and CEP might also be seen as a limitation of our study, and it remains unclear if CEP observed in more homogenous HNSCC patient groups or using the same cutoffs for their definition will be possible. Consequently, we are planning to validate the CEP-risk model in a RCT.

## Conclusions

Whereas individual pre- or post-ICB cytokine concentrations in serum or plasma alone failed to predict the group of patients achieving long-lasting benefit from ICB according to prolonged OS, CEP-risk groups may support their identification. The close link between cytokine expression patterns and modifiable lifestyle-associated risk factors tobacco and alcohol exposure point to their impact on immune surveillance in both the development of HNSCC and response to immune checkpoint blockade.

## Data availability statement

The raw data supporting the conclusions of this article will be made available by the authors, without undue reservation.

## Ethics statement

The studies involving humans were approved by The Institutional Human Ethics Committee of the University Leipzig (vote NICEI-CIH 341-15-ff). The studies were conducted in accordance with the local legislation and institutional requirements. The participants provided their written informed consent to participate in this study.

## Author contributions

Conceptualization: GW. Methodology: GW. Validation: LR, JW, MB, SW, TW, and GW. Formal analysis: LR, TW, and GW. Investigation: LR, IK, VZ, JW, and GW. Resources: AD, VZ, SW, and GW. Data curation: LR, TW, and GW. Writing—original draft preparation: LR and GW. Writing—review and editing: all authors. Visualization: LR and GW. Supervision: SW and GW. Project administration: GW. Funding acquisition: GW and AD. All authors contributed to the article and approved the submitted version.

## Supplementary material

Supplementary Figure 1 (online only). Graphical representation of mean cytokine concentrations measured in serum or EDTA-anticoagulated plasma according to . Error bars indicate 95% confidence intervals. Please note different scaling for individual cytokines named above each plot.
