# Supplementary figures and images for: Immune checkpoint blockade induced shifts in cytokine expression patterns in peripheral blood of head and neck cancer patients are linked to outcome
Source: Front Immunol. 2023 Oct 2;14:1237623. doi: 10.3389/fimmu.2023.1237623 (PMC10577218; doi:10.3389/fimmu.2023.1237623)

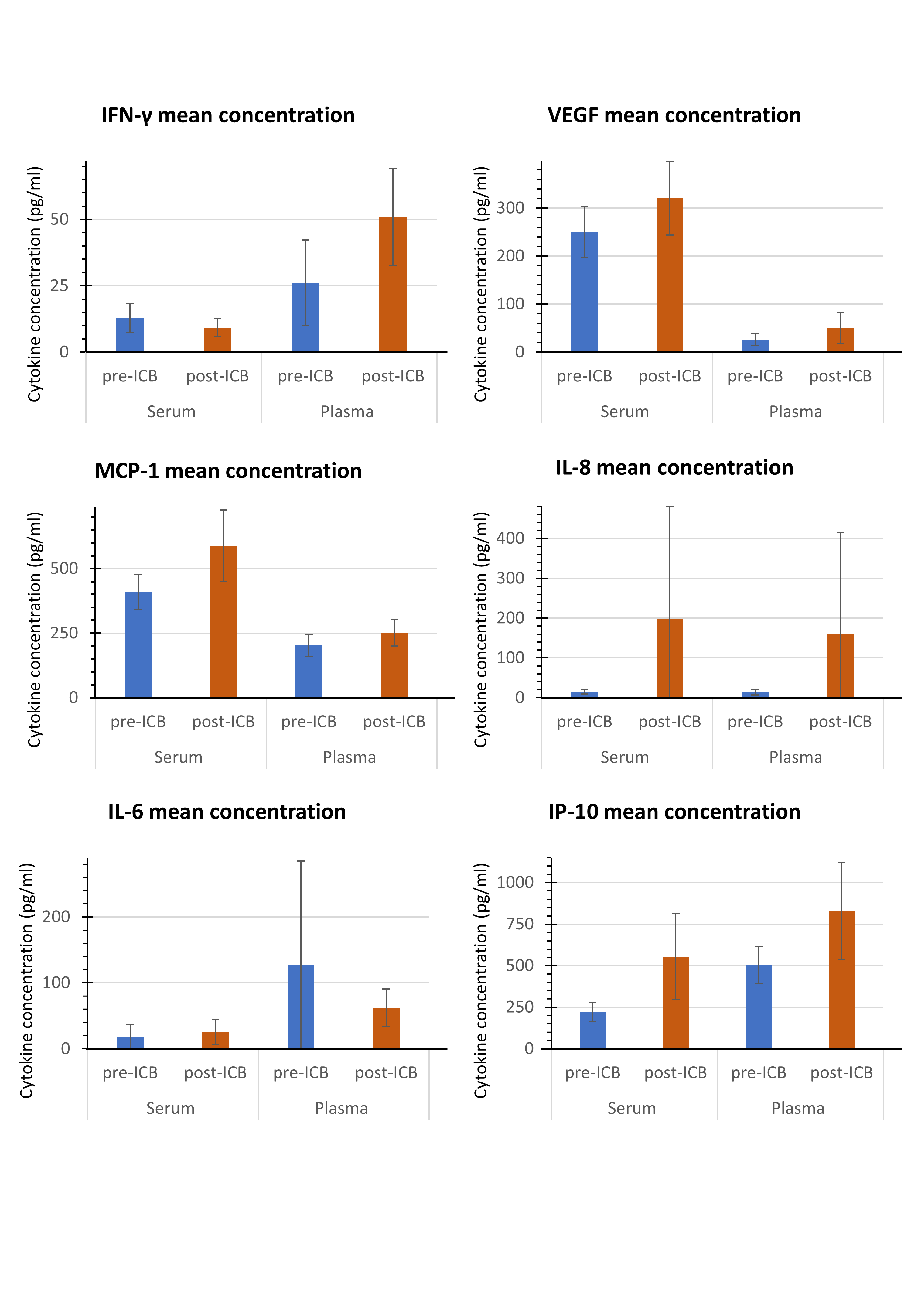

Supplement: Supplementary Figure 1 — (online only). Graphical representation of mean cytokine concentrations measured in serum or EDTA-anticoagulated plasma according to . Error bars indicate 95% confidence intervals. Please note different scaling for individual cytokines named above each plot. [file Image_1.tif]
